# Autophagic activation of IRF‐1 aggravates hepatic ischemia–reperfusion injury via JNK signaling

**DOI:** 10.1002/mco2.58

**Published:** 2021-02-11

**Authors:** Shipeng Li, Jindan He, Hongwei Xu, Jiaxing Yang, Yutian Luo, Wenyue Song, Bingbing Qiao, Haiming Zhang

**Affiliations:** ^1^ Department of Hepatobiliary Surgery First Affiliated Hospital of Xinxiang Medical University Xinxiang China; ^2^ Department of Anesthesiology Peking University Third Hospital Beijing China; ^3^ Department of Obstetrics and Gynecology Jiaozuo Women and Children Hospital Jiaozuo China; ^4^ Department of Hepatobiliary Surgery First Affiliated Hospital of Zhengzhou University Zhengzhou China; ^5^ Department of Liver Transplantation Beijing Friendship Hospital, Capital Medical University Beijing China

**Keywords:** autophagy, hepatic ischemia–reperfusion injury, IRF‐1, JNK

## Abstract

Increasing evidence has accrued indicating that autophagy is associated with hepatic ischemia–reperfusion injury (IRI). This report demonstrates that interferon regulatory factor‐1 (IRF‐1) was upregulated in response to hepatic IRI and was associated with autophagic activation. As a result of these processes, there is an aggravation of liver damage, effects that can be offset by IRF‐1 depletion. In addition, these effects of IRF‐1 are associated with JNK pathway activation followed by increases in Beclin1 protein levels. This JNK‐induced autophagic cell death then leads to cell failure, and plays an important role in liver function damage. We conclude that IRF‐1 activates autophagy through JNK‐mediated autophagy. Accordingly, these findings indicating that the IRF‐1/JNK pathway activates autophagy to exacerbate liver IRI in this mouse model may provide new insights into novel protective therapies for hepatic IRI.

## Graphical abstract

The expression of interferon regulatory factor‐1 (IRF‐1) is related to the high autophagy activity and aggravates the liver damage, which is offset by IRF‐1 depletion in vivo or in vitro. IRF‐1 can activate autophagy to aggravate hepatic ischemia–reperfusion via regulating JNK pathway.

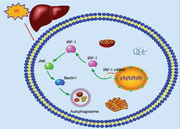

## 1 INTRODUCTION

Hepatic ischemia–reperfusion injury (IRI) is a common condition associated with liver surgery and transplantation.^1^, ^2^, ^3^ Hepatic IRI exerts a critical effect on the outcome of liver surgeries and survival of these patients. Accordingly, it is not surprising that studies directed at exploring the molecular mechanism of liver IRI and the resultant progress of new protection strategy.^4^ However, hepatic IRI involves many factors and complex mechanism, making such investigations problematic.

One of the potential mechanisms associated with IRI is that of autophagy. Autophagy represents a critical process involving tightly regulated intracellular catabolism. As a result, it produces a degradation in cell subsets and lysosomes of proteins and participates in physiological and pathological processes.^5^ Interestingly, autophagy exerts a bidirectional regulatory effect with regard to its role in determining cell survival stage in response to IRI.^5^ Following environmental stress, autophagy can prevent the triggering of apoptosis in cells; however, under long‐term or severe external environmental stimulation, autophagy will lead to cell death.^6^ With regard to ischemia–reperfusion (IR), under conditions of moderate IR treatment, autophagy is upregulated to provide energy for cells,^7^ but with the application of stimulation beyond the tolerance of cells, high levels of autophagy were induced, leading to cell death.^8^ Autophagy is regulated by many molecular processes and signaling pathways, such as autophagy‐related gene (ATG), mTOR, PI3K, and AMPK.^9^, ^10^, ^11^ Recent findings from studies involving tumors, inflammation, and immune diseases have indicated that interferon regulatory factor‐1 (IRF‐1) can participate in the physiological function of cells by activating autophagy. Of particular relevance to the present investigation is the report that levels of IRF‐1 increase within the liver after IR exposure.^12^ Moreover, IRF‐1 knockout, which reduces the expression and release of high mobility group protein (HMGB1), relieves liver IRI.^13^

Despite the potential significance regarding the molecular mechanisms of IRF‐1 in regulating autophagy in hepatic IRI, this area of investigation has received scant attention and therefore further study is sorely needed. In this report, we examined whether the IRF‐1/JNK pathway activates autophagy and aggravates IRI in a mouse model of liver injury. Such findings can serve as the foundation for the development of novel targets in the regulation of autophagy in hepatic IRI.

## 2 MATERIALS AND METHODS

### 2.1 Animals, cell line, and reagents

IRF‐1 WT (IRF‐1^+/+^) and IRF‐1 KO (IRF‐1^−/−^) C57BL/6 mice were purchased from the Casgene Biotechnology Company. All animals were maintained in animal rooms under conditions of controlled temperature, humidity, and photoperiod. The animals received human care with all protocols being in accordance with the guidelines of the National Institutes of Health (NIH) and established standards. AML12 cell line was obtained from ATCC (Virginia, USA). DMEM/F12 medium and fetal bovine serum (FBS) were purchased from GIBCO (Grand Island, New York), and Ad‐IRF‐1 (adenovirus encoding mouse IRF‐1) and Ad‐GFP were purchased from Hanbio. IRF‐1 siRNA and siRNA‐NC were obtained from Ruibo Biological Co., Ltd., (Guangzhou, China). The TUNEL and QRT‐PCR kit were obtained from Roche (Mannheim, Germany), and Lipo 3000 was obtained from Invitrogen (Rockville, Maryland). Antibodies for IRF‐1, p‐JNK, JNK, PCNA, Beclin1, LC3, P62, Bcl‐2, Caspase‐3, Cleave Caspase‐3, and GAPDH were obtained from Cell Signaling Technology Inc. (USA).

### 2.2 Animal model and cell treatment

The hepatic IRI mouse model has been described previously.^3^ For the experiment of adenovirus proliferation, 40 μl Ad‐GFP (1 × 10^10^ PFU) or Ad‐IRF‐1 (1 × 10^10^ PFU) was injected via tail veins. There were 50 mice used in these experiments with 10 in sham treatment group, and 40 mice in the various IRI groups with 10 mice in each group: Ad‐IRF‐1 group, Ad‐GFP group, IRF‐1 siRNA group, and siRNA‐NC group. AML12 cells were divided into six groups: (1) control, nontreated AML12 cells; (2) IR, IRI model as described previously^13^; (3) Ad‐IRF‐1, cells transfected with Ad‐IRF‐1 for 24 h followed by reperfusion; (4) Ad‐GFP, cells transfected with Ad‐GFP followed by reperfusion; (5) IRF‐1 siRNA, cells treated with IRF‐1 siRNA for 24 h prior to reperfusion; and (6) siRNA‐NC, cells treated with siRNA‐NC for 24 h prior to reperfusion.

### 2.3 Serum ALT/AST detection and liver histopathology

Serum levels of alanine aminotransferase (ALT) and aspartate transaminase (AST) in each group were determined with use of kit from the Nanjing Jiancheng Biotechnology Co., Ltd. To assess the extent of injury present, liver tissues were fixed with 4% isotonic formaldehyde for 1 day, dehydrated, embedded in paraffin, and evaluated using HE staining.

### 2.4 Transmission electron microscopy and terminal deoxynucleotidyl transferase dUTP nick‐end labeling

For assessments using transmission electron microscopy (TEM), the cells were placed in 1% glutaraldehyde and fixed with 2% osmium tetroxide. The data were quantified by calculating the number of autophagosomes within a cross‐section area of each cell. Terminal deoxynucleotidyl transferase dUTP nick‐end labeling (TUNEL) was used to evaluate the degree of apoptosis. The average number of TUNEL positive cells in five different visual fields was measured using 100× magnification.

### 2.5 Immunocytochemistry

Immunocytochemistry staining was used to detect protein expression and distributions after microwave treatment. Following incubation in 3% H_2_O_2_ for 10 min, antibodies against PCNA, Bcl‐2, and p‐JNK were added and incubated at 4°C for 12 h. The specimens were incubated with secondary antibodies at 37°C for 1 h, followed by diaminobenzidine staining.

### 2.6 Confocal fluorescent microscopy for detection of autophagy in cells

AML12 cells were transfected with the GFP‐RFP‐LC3 plasmid. DMEM/F12 containing 10% FBS was used instead of medium and cells were treated according to the methods described above. AML12 cells were then washed with PBS and installed with DAPI solution. The cells were then imaged using confocal fluorescence microscopy.

### 2.7 Western blot analysis

Protein samples collected from liver and AML12 cells were transferred from the gel to the nitrocellulose membranes. An identical amount of protein was isolated from SDS polyacrylamide gel at 10%, 12%, or 15%, and detected using antibodies for IRF‐1, p‐JNK, JNK, Beclin1, LC3, P62, Caspase‐3, Cleave Caspase‐3, and GAPDH. Then, ECL assay kit and appropriate HRP‐binding secondary antibodies were used to assess antibody binding. Protein band strengths was standardized according to GAPDH. Each experiment was repeated three times.

### 2.8 Statistical analysis

The data were expressed as means ± standard deviations. Differences among groups were analyzed using one‐way ANOVA and Newman–Keuls test for post hoc *t* pairwise comparisons. The SPSS software 22.0 program was used for these analyses. A *p* < 0.05 was required for results to be considered as statistically significant.

## 3 RESULTS

### 3.1 Serum AST and ALT levels and pathological changes in livers

**FIGURE 1:**
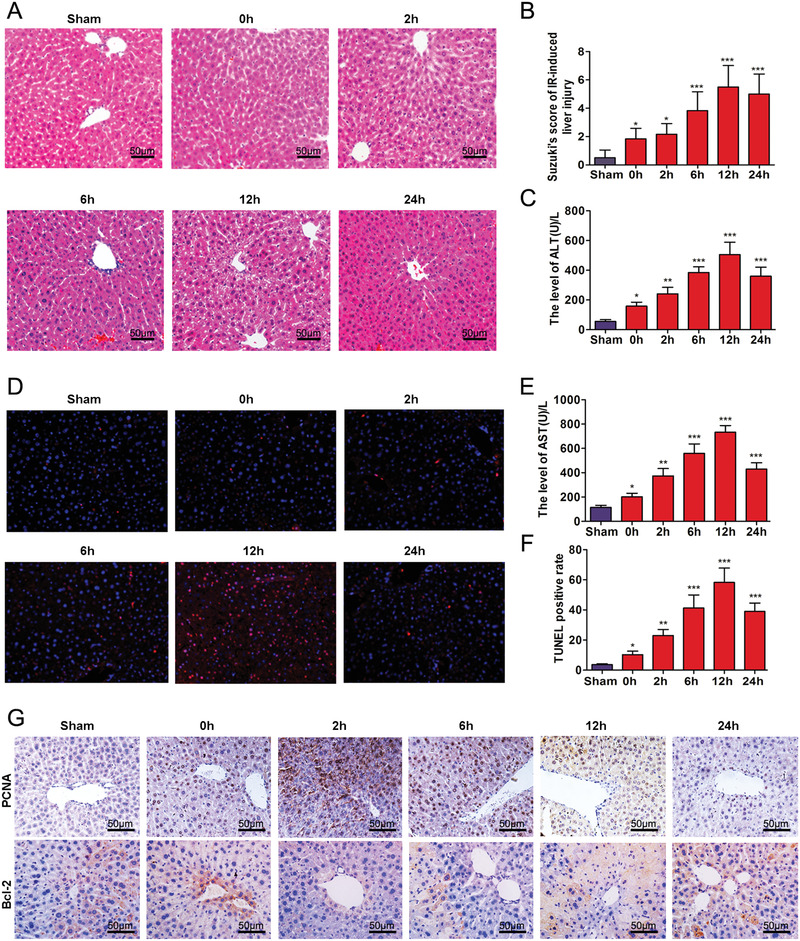
Time‐dependent pathological changes resulting from hepatic IRI in mice. (A) Ischemia, necrosis, and edema as observed at different time points following IRI (scale bars = 50 μm). (B) Suzuki scores in liver IRI. (C and E) Serum ALT and AST levels of mice as a function of increasing reperfusion time. (D) Apoptosis as determined using TUNEL. (F) Analysis of TUNEL positive cells in the liver. (G) Expressions of PCNA and Bcl‐2 in liver as determined using immunocytochemistry (scale bars = 50 μm). ^*^ *p *< 0.05, ^**^ *p* < 0.01, and ^***^ *p* < 0.001 compared with sham group

Results of the pathological analyses are shown in Figure 1A. As compared with that of the sham group, hepatocyte edema, hyperemia, and apoptosis/necrosis were present at 0–24 hours after reperfusion. Simultaneously, there were gradual increases in Suzuki scores (Figure 1B, *p* < 0.05). As shown in Figures 1C and 1E, levels of AST and ALT gradually increased (*p* < 0.05) achieving peak values at 12 h after reperfusion (*p* < 0.001). TUNEL assay results revealed the presence of hepatocyte apoptosis (Figure 1D). As indicated by the red‐colored apoptotic marker, this apoptosis reached peak levels at 12 h after reperfusion (Figure 1F, *p* < 0.05). PCNA and Bcl‐2 protein in hepatic IRI were detected using immunocytochemistry. PCNA protein expression increased at 0–2 h, but decreased at 6–24 h, after reperfusion as compared with that observed in the sham group. Levels of Bcl‐2 increased at 0 h and decreased at 2–24 h after reperfusion (Figure 1G).

### 3.2 IRF‐1, JNK, and autophagy in response to IRI

**FIGURE 2:**
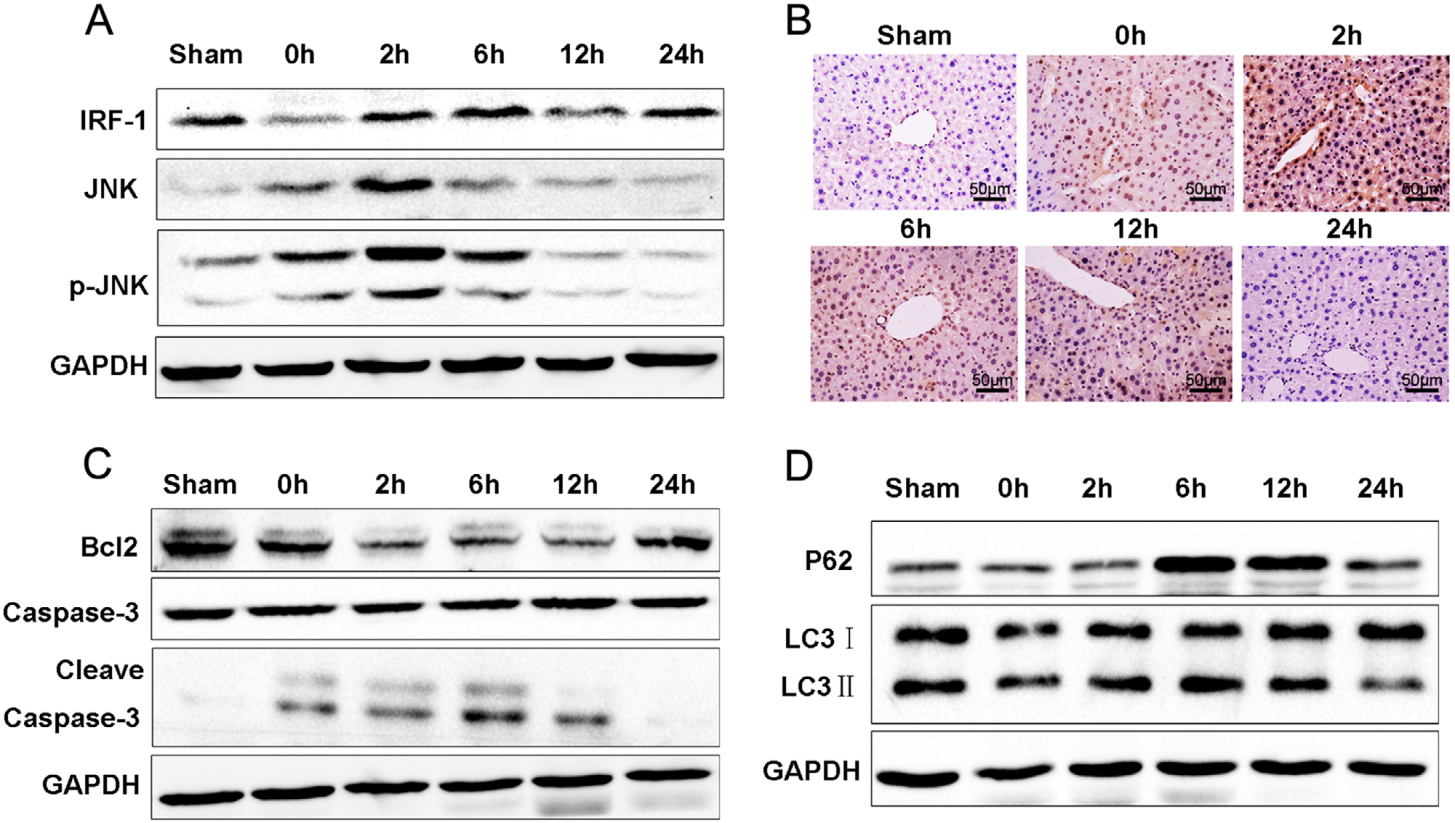
Alterations in IRF‐1 and autophagy in mouse liver IRI. (A) IRI, JNK, and p‐JNK were determined using western blot. Band strength was standardized as based upon the load control of GAPDH. (B) Expressions of p‐JNK in liver as determined using immunocytochemistry (scale bars = 50 μm). (C) Bcl‐2, Caspase‐3, and Cleave Caspase‐3 in livers of sham and IR‐treated mice were determined using immunoblot. Band strength was standardized as based upon the load control of GAPDH. (D) Beclin1, P62, and LC3‐II in livers of sham and IR‐treated mice were determined using immunoblot. Band strength was standardized as based upon the load control of GAPDH

As shown in Figure 2A, compared with that of the sham group, the expression of IRF‐1 was downregulated at following ischemia, whereas IRF‐1 protein expression showed a time‐dependent increased over the 2–24 h period after reperfusion. JNK and p‐JNK expressions were upregulated at 0–2 h after reperfusion peaking at 2 h, followed by a time‐dependent decrease over the 6–24 h period of reperfusion. Similarly, within the liver, protein expression of p‐JNK increased at 0–2 h, but decreased at 6–24 h after reperfusion (Figure 2B). Levels of Bcl‐2 increased at 0 h but decreased significantly at 2–24 h. And the expression of Cleave Caspase‐3 was upregulated at 0–12 h (Figure 2C). Compared with that of the 0 h, the expressions of P62 and LC3‐II protein were increased at 2–12 h after reperfusion (Figure 2D).

### 3.3 IRF‐1 overexpression increases autophagy and hepatic IRI

**FIGURE 3:**
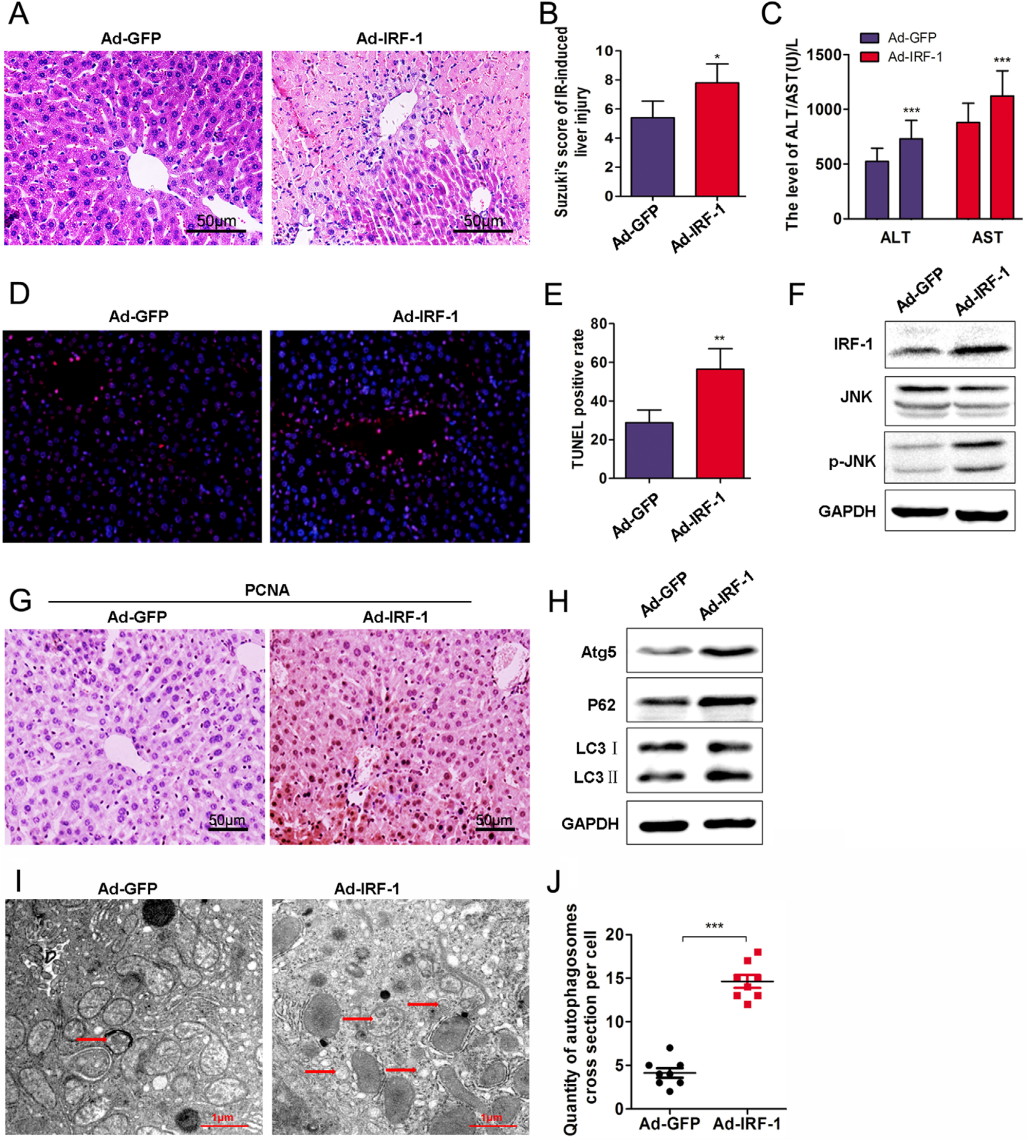
IRF‐1 overexpression increases autophagy and hepatic IRI. (A) Histopathological changes in livers of mice at 12 h after intravenous injection of Ad‐IRF‐1 (scale bars = 50 μm). (B) Suzuki scores in liver IRI. (C) Serum ALT and AST levels in liver IRI. (D) Apoptosis was determined using TUNEL. (E) Analysis of TUNEL positive cells in the liver. (F) IRF‐1, JNK, and p‐JNK were determined using western blot. Band strength was standardized as based upon the load control of GAPDH. (G) Expression of PCNA in mouse liver as determined using immunocytochemistry (scale bars = 50 μm). (H) Atg5, P62, and LC3‐II in the liver of sham and IR‐treated mice were determined using immunoblot. Band strength was standardized as based upon the load control of GAPDH. (I and J) Representative examples of autophagosomes indicated by red arrows in TEM images (scale bars = 1.0 μm). ^*^ *p *< 0.05, ^**^ *p* < 0.01, and ^***^ *p* < 0.001 compared with Ad‐GFP

As shown in Figure 3A, Ad‐IRF‐1 significantly increased the histopathological changes observed in the liver following IR treatment. Suzuki scores were also increased in the Ad‐IRF‐1 group (Figure 3B, *p* < 0.05). As compared with that of the Ad‐GFP group, serum AST and ALT levels in Ad‐IRF‐1 were increased after a prolonged period of reperfusion (Figure 3C, *p* < 0.01). The number of TUNEL positive cells within the Ad‐IRF‐1 group was significantly increased as compared with that of the Ad‐GFP group (Figures 3D and 3E, *p* < 0.01). In addition, IRF‐1 and p‐JNK were all increased in the Ad‐IRF‐1 group (Figure 3F). As compared with that of the Ad‐GFP group, the expression of PCNA (Figure 3G) was significantly increased in the Ad‐IRF‐1 group. Levels of Atg5, P62, and LC3‐II in Ad‐IRF‐1 group were also higher than those in the Ad‐GFP group (Figure 3H). As revealed from TEM (Figure 3I), the number of autophagosomes in the Ad‐IRF‐1 group was significantly increased (Figure 3J, *p* < 0.001).

### 3.4 Effects of IRF‐1 knockout on autophagic signaling and hepatic IRI

**FIGURE 4:**
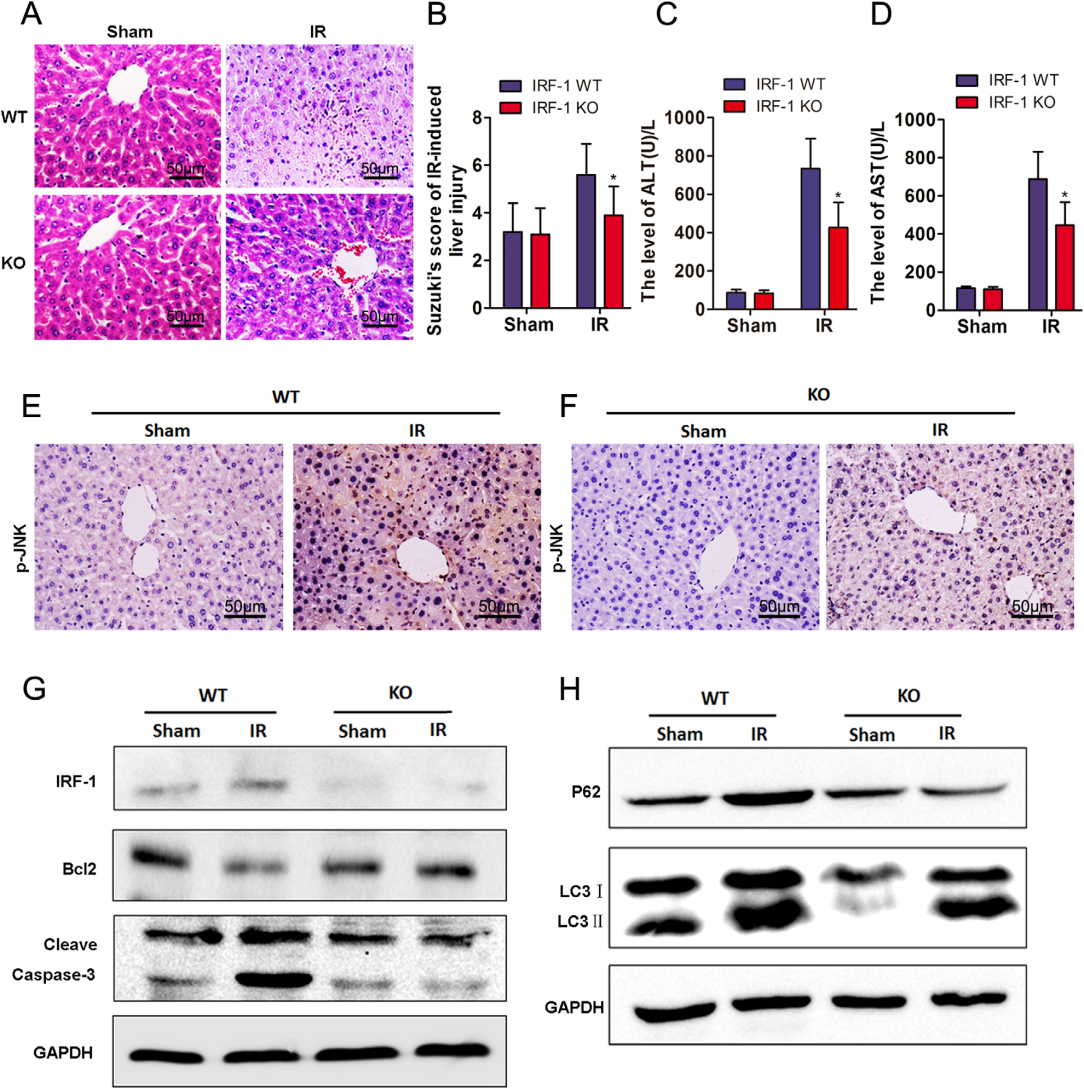
IRF‐1 knockout inhibited autophagy and hepatic IRI. (A) Histopathological changes in livers of WT and IRF‐1 KO mice at 12 h after reperfusion (scale bars = 50 μm). (B) Suzuki scores in liver IRI. (C and D) Serum ALT and AST levels in liver IRI. (E and F) Expression and distribution of p‐JNK as determined using immunocytochemistry (magnification, 200×; scale bars = 50 μm). (G) IRF‐1, Bcl‐2, and Cleave Caspase‐3 levels at 12 h after reperfusion as determined using immunoblot. Band strength was standardized as based upon the load control of GAPDH. (H) P62 and LC3‐II levels at 12 h after reperfusion as determined using western blot. Band strength was standardized as based upon the load control of GAPDH. ^*^ *p* < 0.05 compared with WT

IRF‐1 KO (IRF‐1^−/−)^ mice were used as a means to observe the changes in liver injury and autophagy signals in the absence of IRF‐1. Based on the results obtained with HE staining, here appears to be an improvement in the extent of liver damage resulting from IRI in IRF‐1 KO mice (Figures 4A and 4B). IRF‐1 KO mice exposed to IR showed reductions in liver necrosis, sinusoidal congestion, and ballooning degeneration. When compared to that of IRF‐1 WT mice, serum ALT levels (Figures 4C and 4D, *p* < 0.05) as well as liver p‐JNK at 12 h after IR exposure (Figures 4E and 4F) were significantly decreased in IRF‐1 KO mice. As shown in Figure 4G, expressions of IRF‐1, Cleave Caspase‐3, LC3‐II, and P62 were downregulated, whereas Bcl‐2 was upregulated (Figure 4H).

### 3.5 Autophagic activation by IRF‐1 aggravates AML12 cell IRI

**FIGURE 5:**
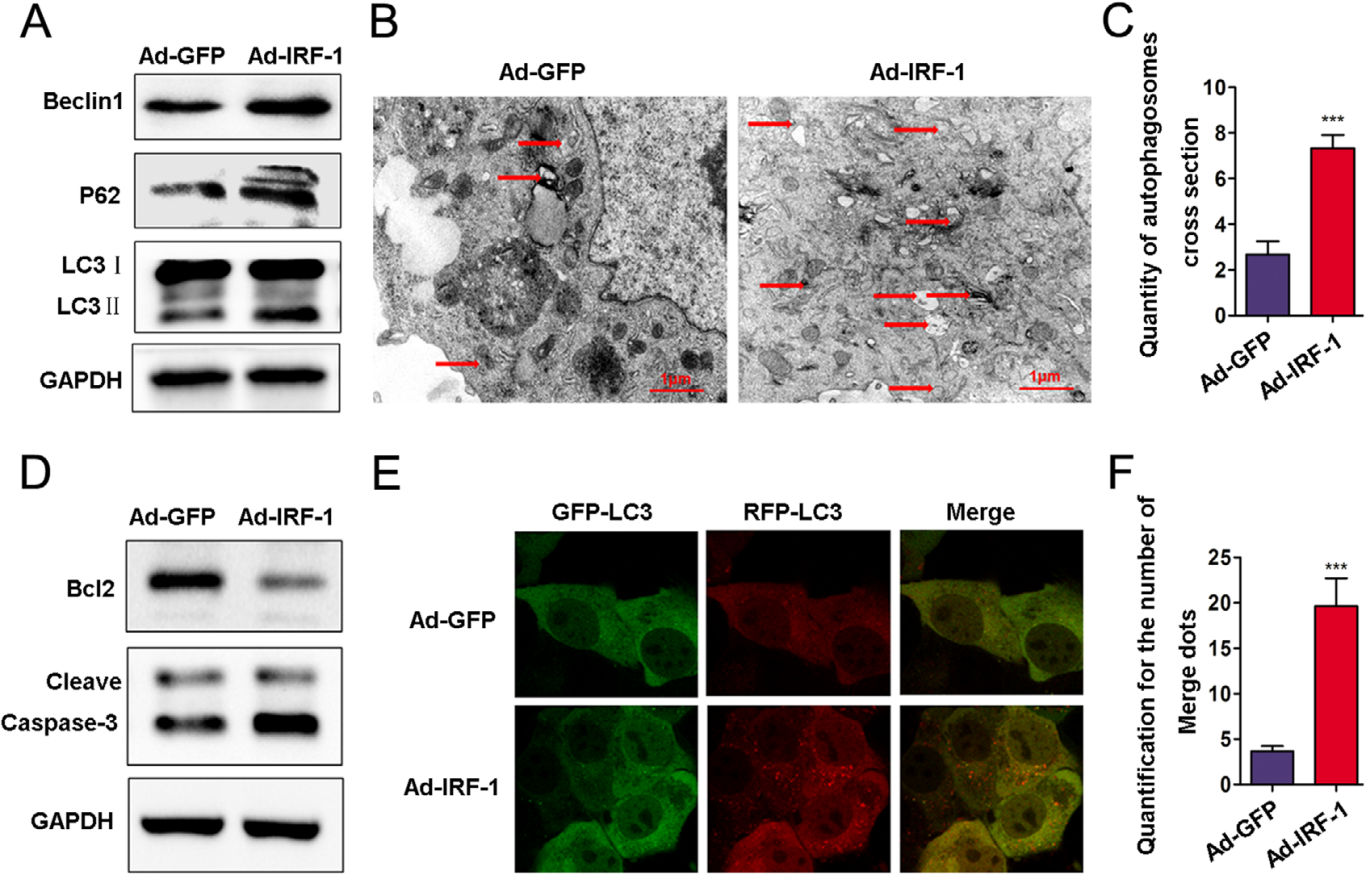
IRF‐1 overexpression increases autophagosomes in AML12 cells. (A) Beclin1, P62, and LC3‐II expressions in AML12 cells treated with Ad‐IRF‐1 as determined using western blot. Band strength was standardized as based upon the load control of GAPDH. (B and C) Representative examples of autophagosomes as indicated by red arrows in TEM images (scale bars = 1.0 μm). (D) Bcl‐2 and Cleave Caspase‐3 in AML12 cells were determined using immunoblot. Band strength was standardized as based upon the load control of GAPDH. (E and F) Confocal immunofluorescence of AML12 cells show that GFP‐RFP‐LC3 spots increased in the Ad‐IRF‐1 group, and both GFP and RFP were expressed in the form of yellow dots (indicating autophagosome) following induction of autophagy. The red dots represent autolysis of GFP in an acidic environment. ^***^ *p* < 0.001 compared with Ad‐GFP

**FIGURE 6:**
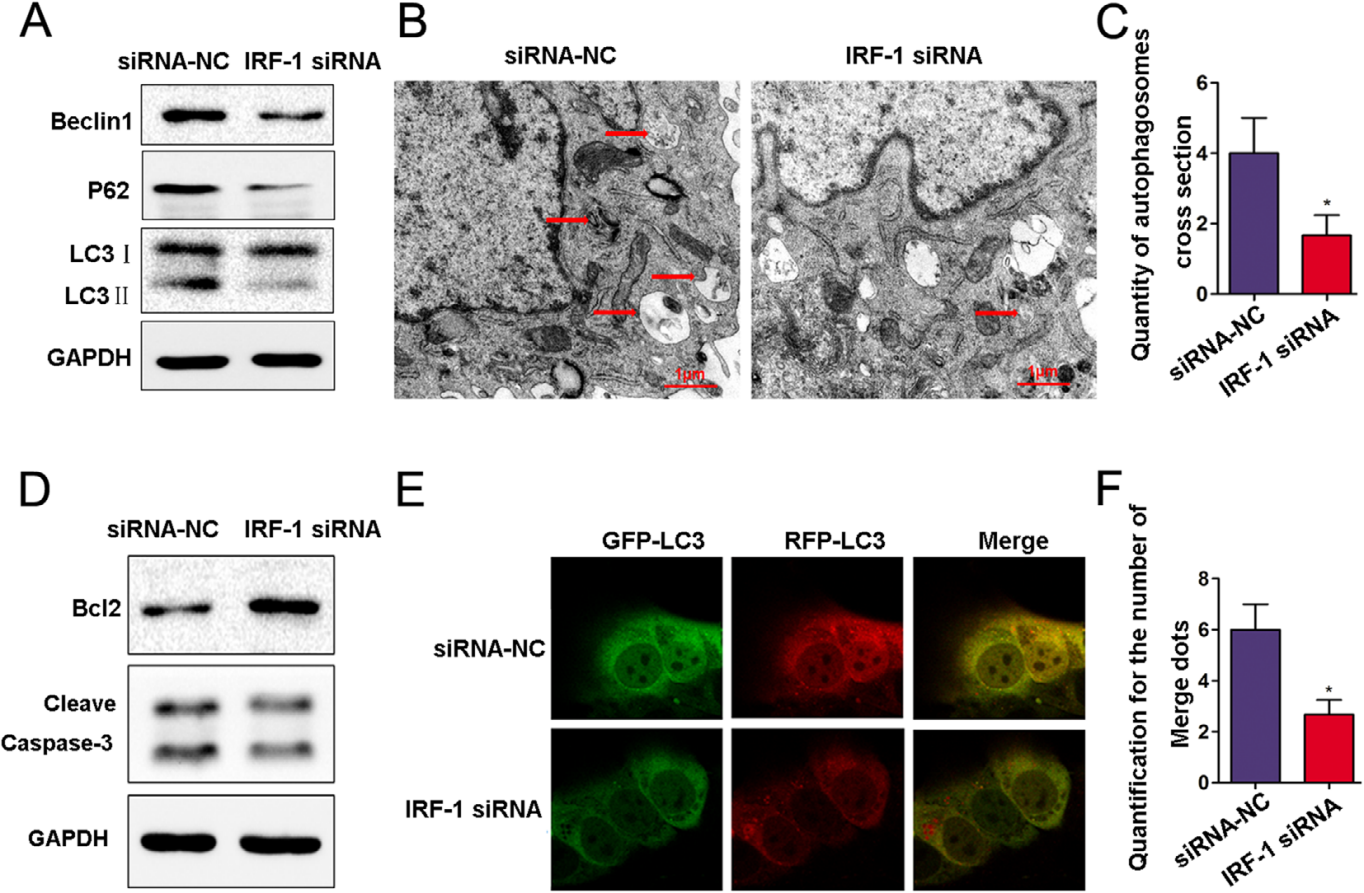
Downregulation of IRF‐1 expression increases autophagosomes in AML12 cells. (A) The levels of Beclin1, P62, and LC3=II in AML12 cells treated with IRF‐1 siRNA as determined using western blot. (B and C) Representative examples of autophagosomes indicated by red arrows in TEM images (scale bars = 1.0 μm). (D) Bcl‐2 and Cleave Caspase‐3 in AML12 cells as determined using immunoblot. (E and F) Confocal immunofluorescence of AML12 cells showing that GFP‐RFP‐LC3 spots increased in the IRF‐1 siRNA group, and both GFP and RFP were expressed in the form of yellow dots (indicating autophagosome) following induction of autophagy. The red dots represent autolysis of GFP in an acidic environment. ^*^ *p* < 0.05 compared with siRNA‐NC

Results from western blot analysis showed that Ad‐IRF‐1 pretreatment enhanced Beclin1, P62, LC3‐II, and Cleave Caspase‐3 as well as increased the levels of Bcl‐2 (Figure 5A and 5D). In contrast, a downregulation of IRF‐1 decreased these levels of Caspase‐3 cleave, Beclin1, P62, and LC3‐II, and significantly decreased the levels of Bcl‐2 (Figures 6A and 6D). TEM was used to evaluate autophagy vacuoles, and autophagy was clearly present (Figures 5B and 5C). Compared with that observed in the Ad‐GFP group, the basic number of autophagosomes in the Ad‐IRF‐1 group was significantly increased and we found that IRF‐1 significantly promoted autophagy. As compared with the Ad‐GFP group, the edge height of yellow and red dots in the Ad‐IRF‐1 group was significantly increased (Figures 5E and 5F). In contrast, a downregulation of IRF‐1 expression significantly reduced autophagy induction (Figures 6B and 6C). Results from confocal fluorescence microscopy showed that compared with the siRNA‐NC group, the marginal eminence of yellow and red dots in the IRF‐1 siRNA group was significantly reduced (Figures 6E and 6F).

## 4 DISCUSSION

The protection of ischemic hepatocytes and reductions in the occurrence of IRI as related to liver donation represent critical issues of vital importance in the field of liver surgery.^14^ Autophagy, which is a process of organelle and protein self‐degradation in cells,^15^ can decompose abnormal proteins and damaged organelles in cells to maintain necessary substrates for cell homeostasis.^16^ Although autophagic activity is upregulated during IR, the relationship between the time course of IR and autophagic activity is controversial.^17^ Findings from some studies suggest that the duration of ischemia or hypoxia is too short to induce changes in autophagy activity, whereas results from other studies indicate that ischemia suppresses and reperfusion upregulates autophagic activity.^18^, ^19^, ^20^ It has even suggested that, in the myocardium, enhancement of autophagy during the ischemic period protects the myocardium, whereas enhancement of autophagy in the reperfusion period damages the myocardium.^8^ Autophagy can clear damaged organelles,^21^ such as mitochondria and endoplasmic reticulum, with the result that the residual organelles cannot meet the needs of cellular activities, leading to apoptosis.^22^, ^23^, ^24^

The findings that expression levels of LC3‐II showed a time‐dependent increase in response to IR time point suggests that IR enhances autophagic activity. We simultaneously analyzed the relationship between levels of autophagic markers and apoptosis of hepatocytes. From this analysis, we found that the expression of LC3 in the early stages of hepatocytes apoptosis in the sham group was less than that in the IRI group, whereas the expression of LC3 and apoptosis from TUNEL assay results were significantly increased in the IRI group. It has recently been reported that IRF‐1 is related in the regulation of autophagy.^25^ For example, Zhang et al.^26^ found that IRF‐1/NO could inhibit autophagy by regulating the growth of hepatoma cells via mTOR.^27^ IRF‐1 promotes liver transplantation IRI through the production of IL‐15/IL‐15Ralpha by hepatocytes. IRF‐1 and IL‐15/IL‐15Ralpha can effectively reduce IRI related to liver transplantation.^28^

In this paper, the effects of IR on autophagy and expression of IRF‐1 were described, with the goal that such information would provide some basis for understanding the role of IRF‐1 in regulating autophagy in liver IRI. Previous work within our laboratory has revealed that IRF‐1 plays a key role in liver IRI by inducing hepatocytes to activate autophagic pathways, in particular by promoting autophagy‐induced cell death through the p38/P62 pathway.^25^ As one approach to assess whether IRF‐1 can aggravate liver IRI, we upregulated the expression of IRF‐1 via tail vein injections of Ad‐IRF‐1 in mice. We found that Ad‐IRF‐1 significantly increased IR‐induced liver histopathological changes. As compared with that of the Ad‐GFP group, the contents of IRF‐1, JNK, p‐JNK, ATG5, P62, and LC3‐II, as well as autophagy, were all increased in the Ad‐IRF‐1 group. To further assess the role of IRF‐1, we examined the changes present in liver injury and autophagic signals in IRF‐1 KO mice. In these mice, we found that IRF‐1, Cleave Caspase‐3, LC3‐II, and P62 were all downregulated, whereas Bcl‐2 was upregulated.

Here, we show that IR can induce autophagy of hepatocytes, aggravate hepatocyte injury, and increase apoptosis. Altogether these effects increase the expression level of IRF‐1, Beclin1, JNK, and p‐JNK protein in response to IR. In conclusion, IR can induce autophagy of mouse hepatocytes, effects that are, in part, related to modulation of autophagy via IRF‐1 and activation of the JNK pathway.

## CONFLICT OF INTEREST

The authors declare no conflict of interest.

## ETHICS APPROVAL

The approval of animal experiments was acquired from the ethics committee of First Affiliated Hospital of Xinxiang Medical University.

## AUTHOR CONTRIBUTION

Shipeng Li, Wenyue Song, Bingbing Qiao, and Haiming Zhang participated in the research design. Jindan He, Jiaxing Yang, and Yutian Luo performed the molecular investigations. Shipeng Li and Jindan He participated in in vivo and in vitro experiment. Jindan He, Jiaxing Yang, and Hongwei Xu performed the data management and statistical analyses after discussion with all authors. Shipeng Li and Jindan He wrote the manuscript.

## Data Availability

All data generated or analyzed during this study are included in this published article.
