# Correction to: Autophagic activation of IRF‐1 aggravates hepatic ischemia–reperfusion injury via JNK signaling

**DOI:** 10.1002/mco2.266

**Published:** 2023-04-26

**Authors:** Shipeng Li, Jindan He, Hongwei Xu, Jiaxing Yang, Yutian Luo, Wenyue Song, Bingbing Qiao, Haiming Zhang

**Affiliations:** ^1^ Department of Hepatobiliary Surgery First Affiliated Hospital of Xinxiang Medical University Xinxiang Henan Province P. R. China; ^2^ Department of Anesthesiology Peking University Third Hospital Beijing P. R. China; ^3^ Obstetrics and Gynecology Jiaozuo Women and Children Hospital Jiaozuo Henan Province P. R. China; ^4^ Department of Hepatobiliary Surgery First Affiliated Hospital of Zhengzhou University Zhengzhou Henan Province P. R. China; ^5^ Department of Liver Transplantation Beijing Friendship Hospital Capital Medical University Beijing PR China

Shipeng Li^1,*,#^, Jindan He^2,#^, Hongwei Xu^1^, Jiaxing Yang^1^, Yutian Luo^1^, Wenyue Song^3^, Bingbing Qiao^4,*^, Haiming Zhang^5,*^

^1^Department of Hepatobiliary Surgery, First Affiliated Hospital of Xinxiang Medical University, Xinxiang, Henan Province, P. R. China

^2^Department of Anesthesiology, Peking University Third Hospital, Beijing, P. R. China

^3^Obstetrics and Gynecology, Jiaozuo Women and Children Hospital, Jiaozuo, Henan Province, China

^4^Department of Hepatobiliary Surgery, First Affiliated Hospital of Zhengzhou University, Zhengzhou, Henan Province, P. R. China

^5^Department of Liver Transplantation, Beijing Friendship Hospital, Capital Medical University, Beijing, PR China

^#^Co‐first authors: Shipeng Li and Jindan He

^*^ **Correspondence Author**:

**Shipeng Li**, Department of Hepatobiliary Surgery, First Affiliated Hospital of Xinxiang Medical University, Xinxiang, Henan Province, 453100, P. R. China. Email: shipengli2010@163.com

**Bingbing Qiao**, Department of Hepatobiliary Surgery, First Affiliated Hospital of Zhengzhou University, Zhengzhou, Henan Province, 450000, P. R. China. Email: popzxcbb@126.com

**Haiming Zhang**, Department of Liver Transplantation, Beijing Friendship Hospital, Capital Medical University, Beijing 100050, PR China. Email: mdzhanghaiming@qq.com

First published: 11 February 2021, https://doi.org/10.1002/mco2.58

In the process of checking the raw data^1^, the authors noticed several inadvertent mistakes occurring in Figure 1D, Figure 3A/F/I, Figure 5B and Figure 6C during the preparation of these panels. The correct results should be as shown below. The authors apologize for these oversights and declare that these corrections do not affect the description, interpretation, or conclusions detailed in the original manuscript.

**FIGURE 1:**
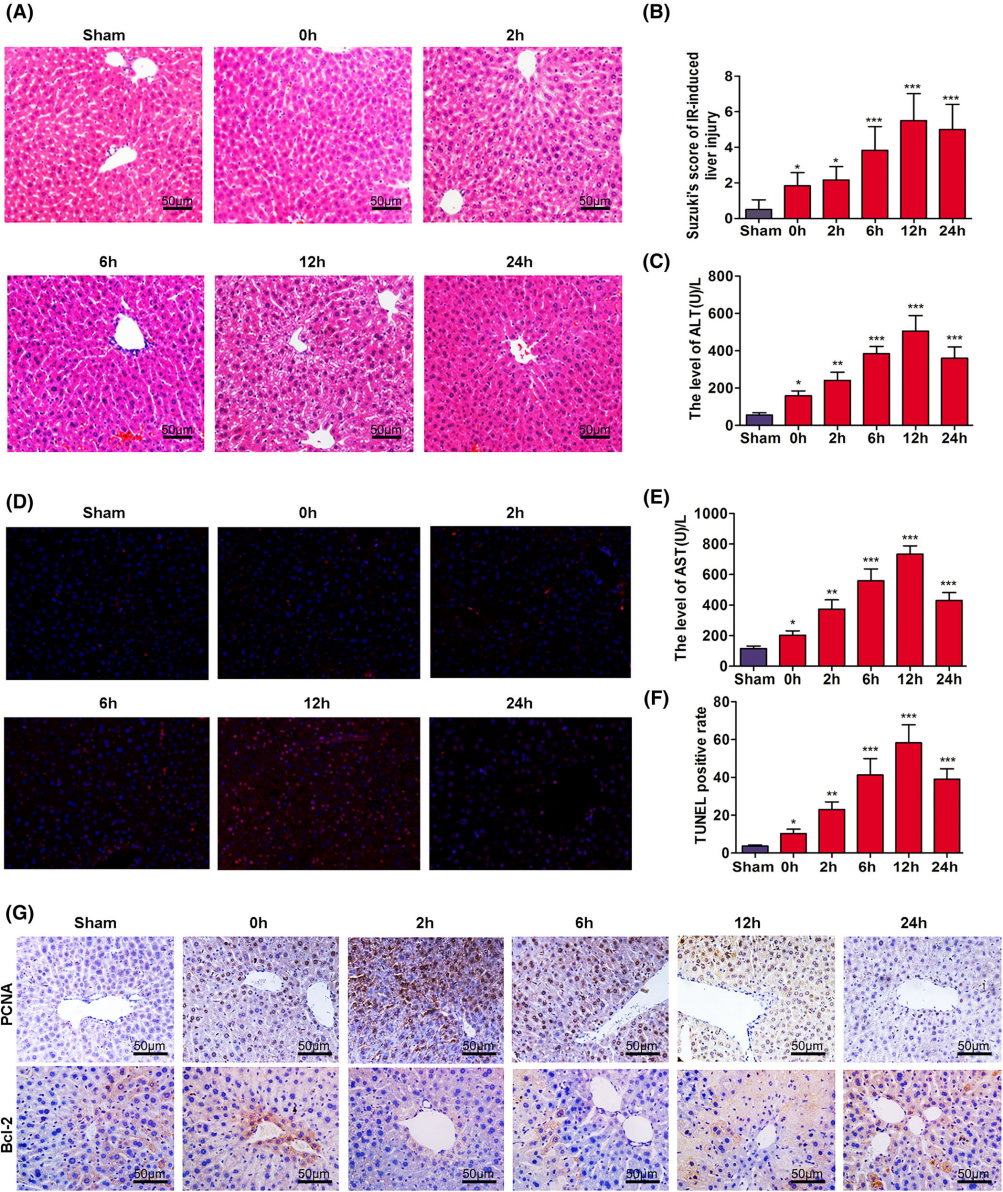
Time‐dependent pathological changes resulting from hepatic IRI in mice. (A) Ischemia, necrosis, and edema as observed at different time points following IRI (scale bars = 50 μm). (B) Suzuki scores in liver IRI. (C and E) Serum ALT and AST levels of mice as a function of increasing reperfusion time. (D) Apoptosis as determined using TUNEL. (F) Analysis of TUNEL positive cells in the liver. (G) Expressions of PCNA and Bcl‐2 in liver as determined using immunocytochemistry (scale bars = 50 μm). *p < 0.05, **p < 0.01, and ***p < 0.001 compared with sham group

**FIGURE 3:**
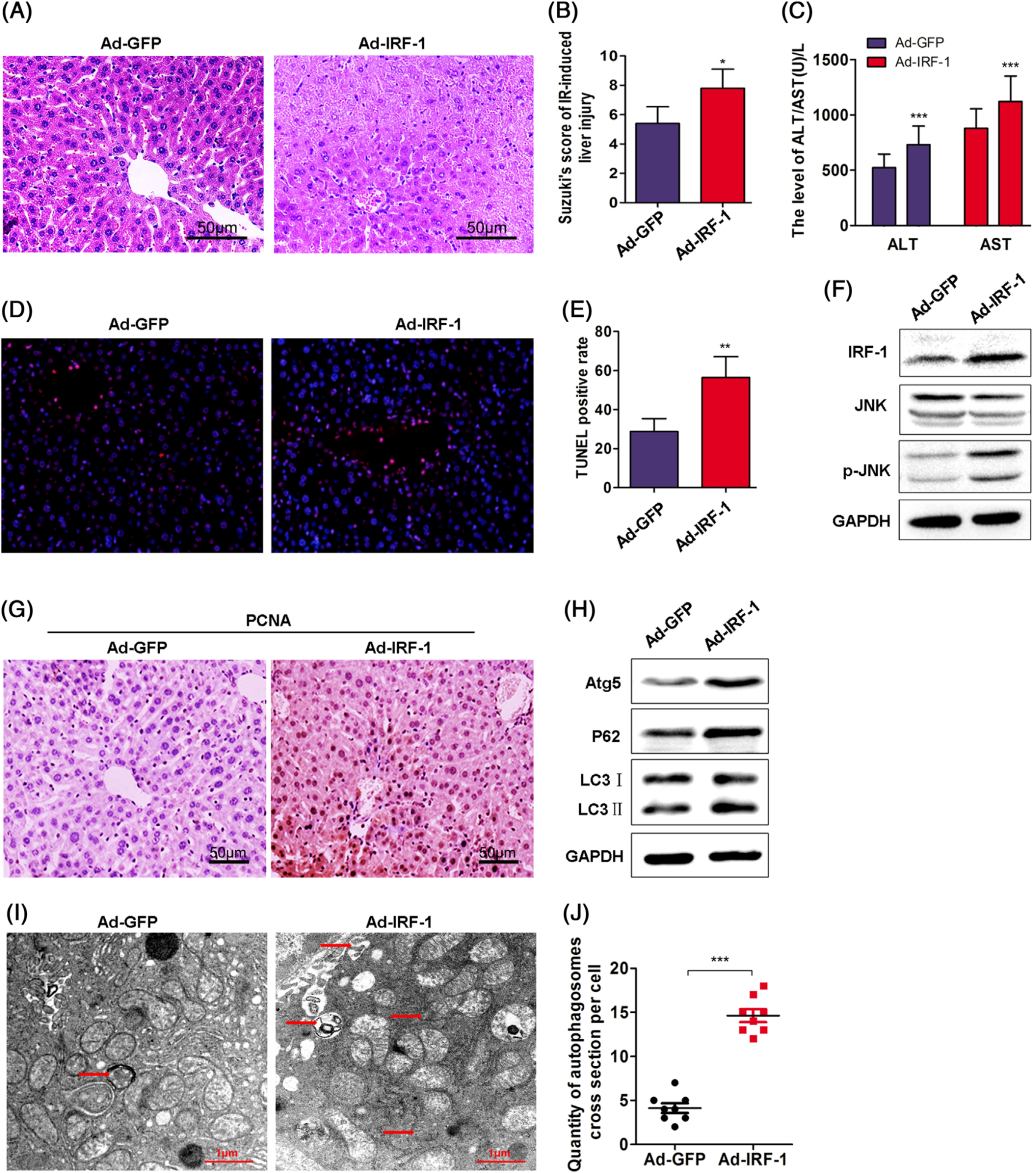
IRF‐1 overexpression increases autophagy and hepatic IRI. (A) Histopathological changes in livers of mice at 12 h after intravenous injection of Ad‐IRF‐1 (scale bars = 50 μm). (B) Suzuki scores in liver IRI. (C) Serum ALT and AST levels in liver IRI. (D) Apoptosis was determined using TUNEL. (E) Analysis of TUNEL positive cells in the liver. (F) IRF‐1, JNK, and p‐JNK were determined using western blot. Band strength was standardized as based upon the load control of GAPDH. (G) Expression of PCNA in mouse liver as determined using immunocytochemistry (scale bars = 50 μm). (H) Atg5, P62, and LC3‐II in the liver of sham and IR‐treated mice were determined using immunoblot. Band strength was standardized as based upon the load control of GAPDH. (I and J) Representative examples of autophagosomes indicated by red arrows in TEM images (scale bars = 1.0 μm). *p < 0.05, **p < 0.01, and ***p < 0.001 compared with Ad‐GFP

**FIGURE 5:**
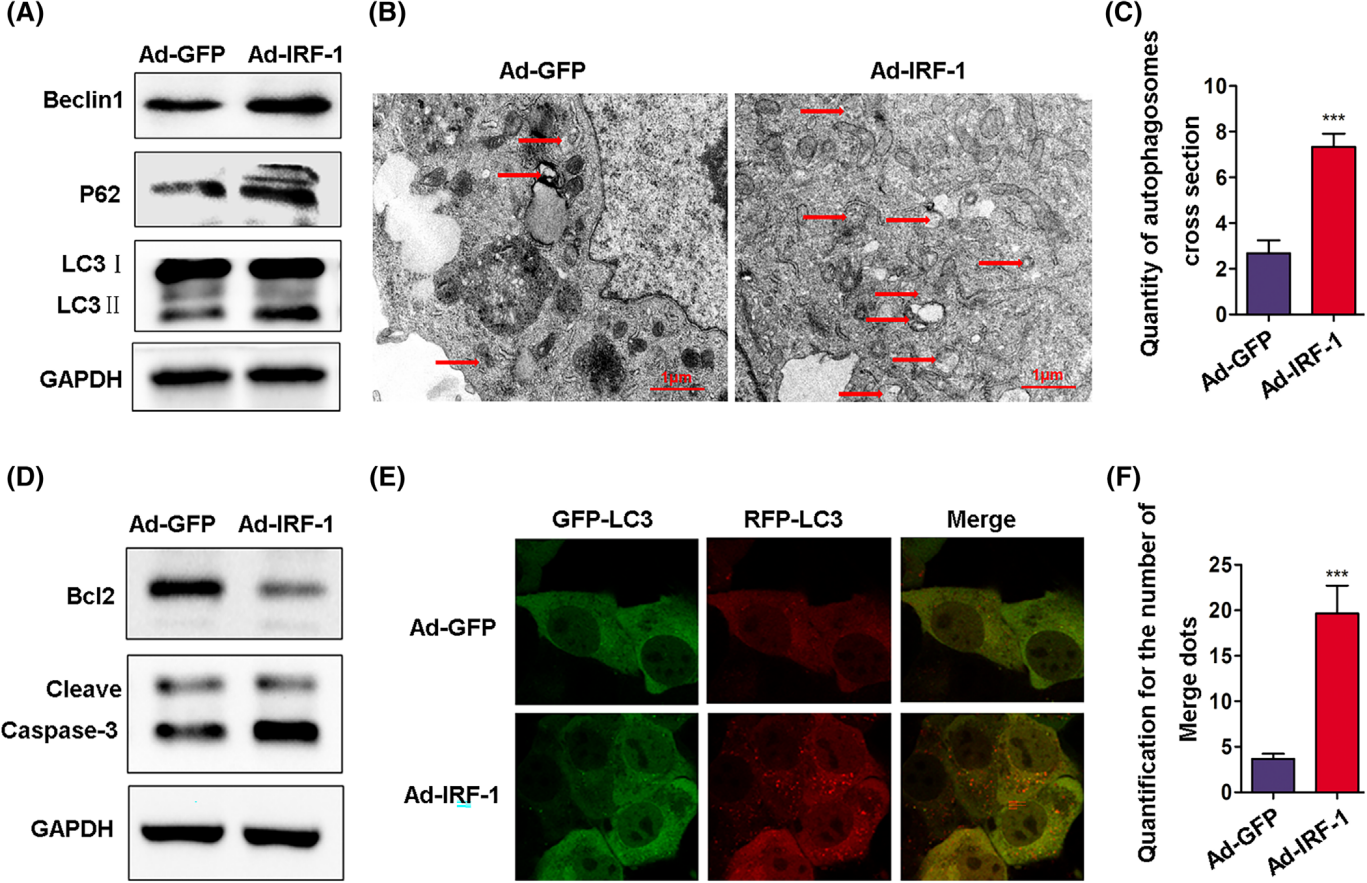
IRF‐1 overexpression increases autophagosomes in AML12 cells. (A) Beclin1, P62, and LC3‐II expressions in AML12 cells treated with Ad‐IRF‐1 as determined using western blot. Band strength was standardized as based upon the load control of GAPDH. (B and C) Representative examples of autophagosomes as indicated by red arrows in TEM images (scale bars = 1.0 μm). (D) Bcl‐2 and Cleave Caspase‐3 in AML12 cells were determined using immunoblot. Band strength was standardized as based upon the load control of GAPDH. (E and F) Confocal immunofluorescence of AML12 cells show that GFP‐RFP‐LC3 spots increased in the Ad‐IRF‐1 group, and both GFP and RFP were expressed in the form of yellow dots (indicating autophagosome) following induction of autophagy. The red dots represent autolysis of GFP in an acidic environment. ***p < 0.001 compared with Ad‐GFP

**FIGURE 6:**
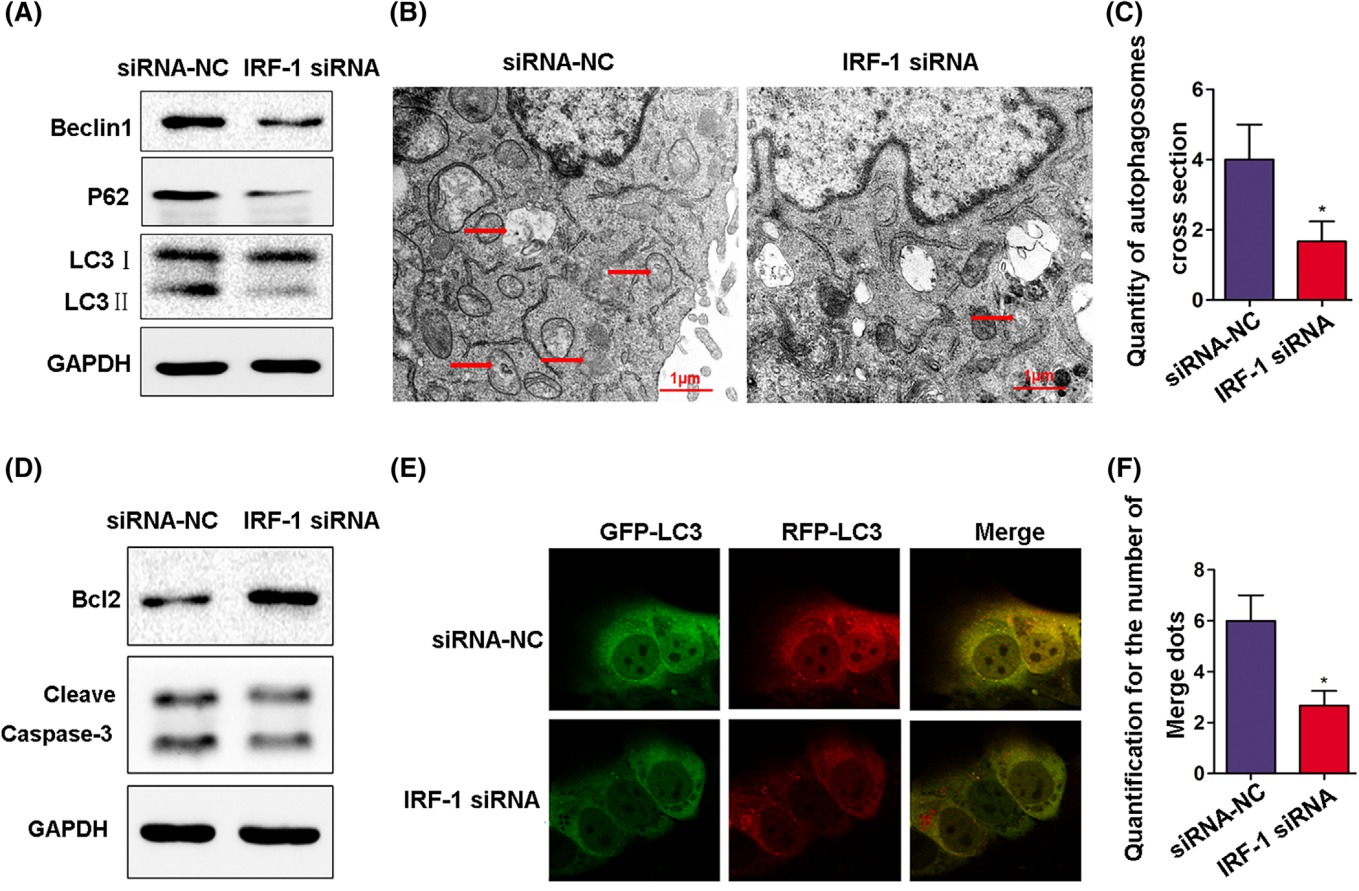
Downregulation of IRF‐1 expression increases autophagosomes in AML12 cells. (A) The levels of Beclin1, P62, and LC3 = II in AML12 cells treated with IRF‐1 siRNA as determined using western blot. (B and C) Representative examples of autophagosomes indicated by red arrows in TEM images (scale bars = 1.0 μm). (D) Bcl‐2 and Cleave Caspase‐3 in AML12 cells as determined using immunoblot. (E and F) Confocal immunofluorescence of AML12 cells showing that GFP‐RFP‐LC3 spots increased in the IRF‐1 siRNA group, and both GFP and RFP were expressed in the form of yellow dots (indicating autophagosome) following induction of autophagy. The red dots represent autolysis of GFP in an acidic environment. *p < 0.05 compared with siRNA‐NC

**Abbreviations**: IRI, ischemia‐reperfusion injury; ALT, alanine transaminase; AST, aspartate transaminase; TUNEL, Terminal Deoxynucleotidyl Transferase Mediated dUTP Nick End Labeling; PCNA, proliferating cell nuclear antigen; IRF, Interferon Regulatory Factor; JNK, c‐Jun N‐terminal kinase; GAPDH, glyceraldehyde‐3‐phosphate dehydrogenase; GFP, green fluorescent protein; TEM, Transmission Electron Microscope.
